# A Curious Case of Acute Respiratory Failure: Is It Antisynthetase Syndrome?

**DOI:** 10.1155/2016/7379829

**Published:** 2016-06-28

**Authors:** Gurveen Malhotra, Nitya Ramreddy, Serafin Chua, Mira Iliescu, Tanjeev Kaur

**Affiliations:** ^1^University of Illinois at Chicago, Chicago, IL 60612, USA; ^2^Internal Medicine, Mount Sinai Hospital, Chicago, IL 60608, USA; ^3^Rheumatology, Mount Sinai Hospital, Chicago, IL 60608, USA; ^4^Pulmonary & Critical Care Medicine, Mount Sinai Hospital, Chicago, IL 60608, USA

## Abstract

Antisynthetase (AS) syndrome is a major subgroup of inflammatory myopathies seen in a minority of patients with dermatomyositis and polymyositis. Although it is usually associated with elevated creatine phosphokinase level, some patients may have amyopathic dermatomyositis (ADM) like presentation with predominant skin involvement. Interstitial lung disease (ILD) is the main pulmonary manifestation and may be severe thereby determining the prognosis. It may rarely present with a very aggressive course resulting in acute respiratory distress syndrome (ARDS). We report a case of a 43-year-old male who presented with nonresolving pneumonia who was eventually diagnosed to have ADM through a skin biopsy without any muscle weakness. ADM may be associated with rapidly progressive course of interstitial lung disease (ADM-ILD) which is associated with high mortality. Differentiation between ADM-ILD and AS syndrome may be difficult in the absence of positive serology and clinical presentation may help in clinching the diagnosis.

## 1. Introduction

Antisynthetase (AS) syndrome is a rare disease entity with predominant interstitial lung disease associated with the presence of anti-Jo-1 antibodies. It may pose a diagnostic challenge and needs early aggressive immunosuppression for better outcomes.

## 2. Case Presentation

A 43-year-old male without any past medical history presented with progressive dyspnea, fever, and nonproductive cough for 1 month. He denied orthopnea, paroxysmal nocturnal dyspnea, hemoptysis, or weight loss. There was no history of recent travel, sick contacts, or prolonged immobilization. He had no history of cigarette smoking, second-hand smoking, or alcohol or illicit drug use.

He was afebrile and hemodynamically stable on presentation. He had mild periorbital edema with erythema on supraorbital areas but no heliotrope rash. He also had erythematous patchy scaly lesions on bilateral elbows and metacarpophalangeal joints with periungual erythema without distinct Gottron papules. Breath sounds were decreased on the right base with crackles and scattered rhonchi. There was no gallop rhythm or jugular venous distention. Mildly tender metacarpophalangeal and proximal interphalangeal joints without synovitis were noted bilaterally. Muscle strength was normal and equal bilaterally in all four extremities. Initial blood work showed aspartate transaminase 94 U/L, alanine transaminase 70 U/L, and no leucocytosis.

**Figure 1:**
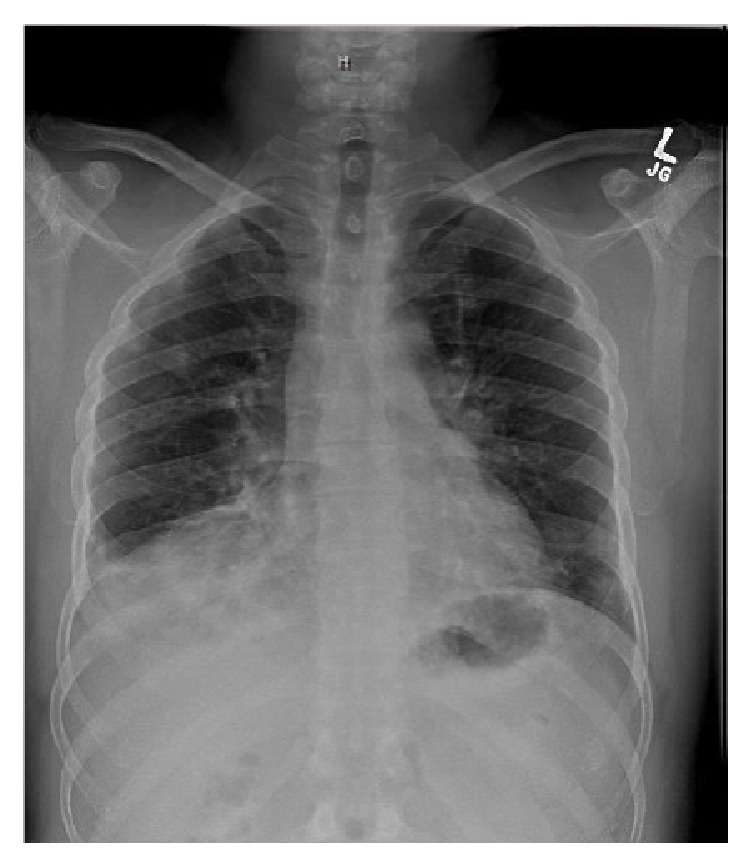
Chest X-ray showing bibasilar and right middle lobe infiltrate on presentation.

**Figure 2:**
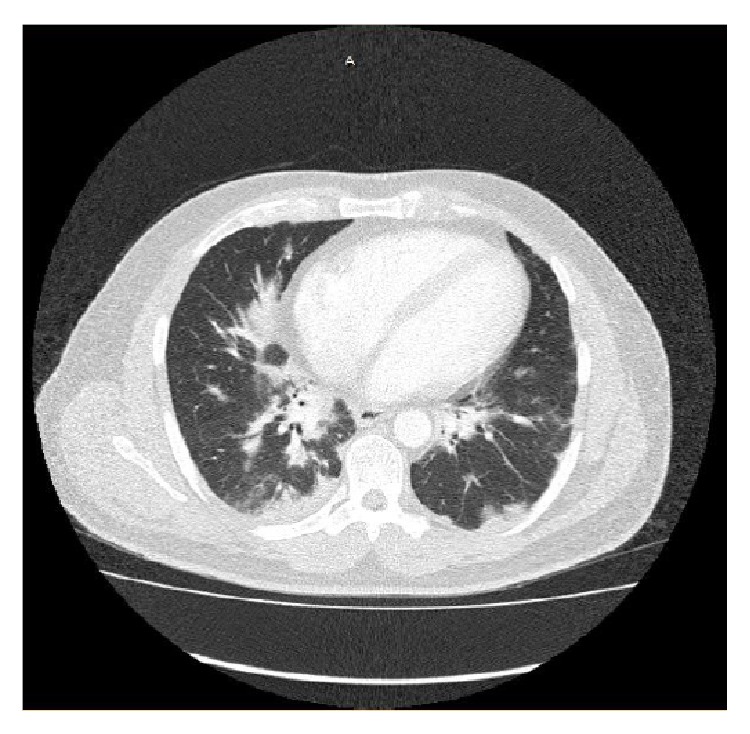
CT chest revealing bilateral basilar fibrosis along with reticulonodular and subpleural wedge shaped opacities.

**Figure 3:**
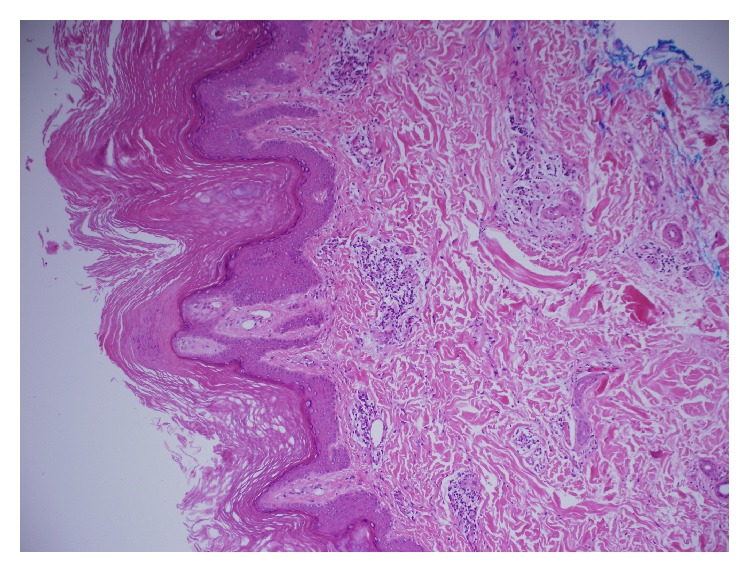
Skin biopsy shows acanthosis and papillomatosis with focal dermal perivascular lymphocytic infiltrate (10x).

He later developed a fever and chest X-ray (Figure 1) was done which revealed bilateral lower and right middle lobe infiltrates. Respiratory panel, blood culture, sputum culture, fungal culture,* Legionella* antigen,* Histoplasma* antigen, QuantiFERON Gold, hepatitis panel, and HIV test were all negative. A computed tomography scan of the chest (Figure 2) showed patchy subpleural bilateral opacities more prominent in the bases with subtle reticulonodular opacities in both lungs along with traction bronchiectasis. No significant pleural effusion, pneumothorax, or lymphadenopathy was seen. He was recently treated for pneumonia at another hospital and was thus empirically started on antibiotics for treatment of suspected health care associated pneumonia. Bronchoscopy with transbronchial biopsy revealed chronic inflammation and fibrosis. Autoimmune workup including anti-cyclic citrullinated peptide, anti-nuclear antibody, double-stranded DNA antibody, anti-proteinase 3, anti-myeloperoxidase, Scl-70, anti-smooth muscle, anti-Jo-1 antibody, and cryoglobulin was unremarkable. Aldolase was elevated at 13.7 (normal < 8.1 U/L), C-reactive protein was 10.4 (normal 0–8 mg/L), creatine phosphokinase was 351 (normal 30–223 IU/L), and rheumatoid factor was 71 (normal < 14 IU/mL). Skin biopsy of lesions over metacarpophalangeal joints revealed mild epidermal acanthosis and papillomatosis with perivascular inflammation suggestive of dermatomyositis (Figure 3).

Pulse dose of intravenous methylprednisolone 1000 mg was given for 3 days. Video assisted thoracoscopic lung biopsy could not be performed because of his worsening respiratory status. The clinical picture with the presence of ILD, fever, and arthritis in the setting of ADM suggested a diagnosis of AS syndrome and immunosuppression with cyclophosphamide was initiated. Although he was initially placed on noninvasive ventilation, his condition eventually deteriorated requiring intubation and mechanical ventilation for hypoxemic respiratory failure. Repeat chest X-ray showed findings consistent with acute respiratory distress syndrome (ARDS) and he was placed on RotoProne bed. Repeat creatine kinase level was minimally elevated at 292 IU/L and urinalysis revealed red blood cell casts. He subsequently developed acute kidney injury with metabolic acidosis requiring hemodialysis. He eventually went into shock requiring vasopressor support and progressed to multiorgan failure. His condition continued to deteriorate leading to refractory shock with no improvement in the overall status; the family decided not to resuscitate in the event of a cardiac arrest and he unfortunately died.

## 3. Discussion

Inflammatory myopathies are a diverse group of autoimmune disorders with polymyositis, dermatomyositis, and inclusion body myositis as the major entities. Skin disease may precede the development of myopathy while in others the disease only affects the skin, ADM.

A major subgroup of inflammatory myopathies are AS syndrome characterized by ILD, chronic polyarthritis, Raynaud's phenomenon, fever, and “mechanics” hands [1]. Diagnosis is confirmed by the presence of anti-tRNA synthetase autoantibodies with anti-Jo-1 antibody being the most common [2]. Other less commonly tested antibodies include anti-PL-7 (anti-threonyl), anti-PL-12 (anti-alanyl), and anti-tyrosyl tRS antibody which are found in only 2–5% of the cases [3]. The true incidence of AS syndrome is unknown; however, the annual incidence of inflammatory myopathies ranges between 2 and 10 cases per million per year [4] and antisynthetase antibodies are found in 25–40% of them [5]. Interestingly, AS syndrome is very infrequently seen in ADM [6].

Lung involvement is rare in ADM with nonspecific interstitial pneumonia being the most common pattern [6] and associated with poor prognosis [7, 8]. Other presentations may include diffuse alveolar damage, usual interstitial pneumonia, cryptogenic organizing pneumonia, and ARDS [9]. Although data on ILD in ADM is not very clear, two different forms have been established: acute and chronic [10]. The acute subtype is more prevalent in the amyopathic type as compared to the classic dermatomyositis and associated with much higher mortality. Acute ILD rapidly deteriorates into respiratory failure which is the leading cause of death in these patients. The presence of traction bronchiectasis may help in differentiating rapidly deteriorating subtype from the chronic form and hence determining the prognosis [11]. The acute form of ILD is also associated with a high mortality of up to 67% when compared to the chronic form [8].

There is no current established treatment but the mainstay of therapy has been high dose steroids and immunosuppressive agents like cyclophosphamide and cyclosporine [2, 12]. In resistant cases, tacrolimus [13], intravenous immunoglobulin, and rituximab have shown benefit [14].

Kidney injury is infrequent in ADM. Rhabdomyolysis and myoglobulinuria leading to acute tubular necrosis are the most common cause while glomerulonephritis is less frequently seen [15]. The major glomerulopathies are membranous nephritis, membranoproliferative glomerulonephritis, and diffuse proliferative glomerulonephritis. The serological tests for various vasculitides as well as the viral hepatitis were all negative. Our patient could not get a kidney biopsy because of his hemodynamically unstable condition.

It is therefore a challenge to diagnose AS syndrome in the absence of confirmatory serological tests, and, in such a scenario, ADM-ILD should be considered. Diagnosis can be made in similar situations after ruling out various connective tissue diseases as the primary etiology. The clinical scenario presented by the authors here emphasizes the need for physicians to become familiar with this clinical entity of AS syndrome since early diagnosis and treatment usually lead to better outcomes. A delay in the diagnosis as in our patient may result in severe complications and a poor prognosis despite aggressive treatment. Further, management may be difficult and requires corticosteroids and immunosuppression in either situation. Both AS syndrome and ILD can rapidly deteriorate into acute respiratory failure which may prove fatal.
